# Maternal Prepregnancy Body Mass Index and Gestational Weight Gain on Offspring Overweight in Early Infancy

**DOI:** 10.1371/journal.pone.0077809

**Published:** 2013-10-11

**Authors:** Nan Li, Enqing Liu, Jia Guo, Lei Pan, Baojuan Li, Ping Wang, Jin Liu, Yue Wang, Gongshu Liu, Gang Hu

**Affiliations:** 1 Tianjin Women’s and Children’s Health Center, Tianjin, China; 2 Chronic Disease Epidemiology Laboratory, Pennington Biomedical Research Center, Baton Rouge, Louisiana, United States of America; Sookmyung Women's University, Korea, Republic Of

## Abstract

**Objective:**

The aim of the present study was to evaluate the association of maternal prepregnancy body mass index (BMI) and gestational weight gain (GWG) with anthropometry in the offspring from birth to 12 months old in Tianjin, China.

**Methods:**

Between 2009 and 2011, health care records of 38,539 pregnant women had been collected, and their children had been measured body weight and length at birth, 3, 6, 9 and 12 months of age. The independent and joint associations of pre-pregnancy BMI and GWG based on the Institute of Medicine (IOM) guidelines with anthropometry in the offspring were examined using General Linear Model and Logistic Regression.

**Results:**

Prepregnancy BMI and maternal GWG were positively associated with Z-scores for birth weight-for-gestational age, birth length-for-gestational age, and birth weight-for-length. Infants born to mothers with excessive GWG had the greatest changes in Z-scores for weight-for-age from birth to Month 3, and from Month 6 to Month 12, and the greatest changes in Z-scores for length-for-age from birth to months 3 and 12 compared with infants born to mothers with adequate GWG. Excessive GWG was associated with an increased risk of offspring overweight or obesity at 12 months old in all BMI categories except underweight.

**Conclusions:**

Maternal prepregnancy overweight/obesity and excessive GWG were associated with greater weight gain and length gain of offspring in early infancy. Excessive GWG was associated with increased infancy overweight and obesity risk.

## Introduction

Improvements of maternal, fetal, and child health are key public health goals. Childhood obesity is a global problem. Worldwide, the childhood obesity prevalence in 2010 is 6.7%, and 70% of obese adolescents become obese adults [1]. Developing effective prevention and intervention programs for the children at formative pre-school years (2-6 years old) might be an important step in combating the childhood obesity epidemic. Some studies have indicated that higher birth weight may be a risk factor of obesity in the late life [2-4].

In recent years, maternal prepregnancy body mass index (BMI) has increased among the childbearing age women in developed countries [5]. The Pregnancy Nutrition Surveillance System (PNSS) reported that approximately one-half of women gained more weight than recommended by the Institute of Medicine (IOM) guidelines. In the 2009 PNSS, 21.2% of women gained less weight than recommended during pregnancy, 30.6% gained the recommended amount of weight, and 48.2% gained more weight than recommended [6]. Moreover, women who are overweight or obese at the start of pregnancy or gain weight excessively or inadequately during pregnancy are at increased risk of poor maternal and child health outcomes. Several recent studies reported that prepregnancy BMI was positively associated with infant birth weight [7,8], and excessive gestational weight gain (GWG) was associated with many pregnancy complications such as preterm birth, cesarean delivery, and large for gestational age neonates [9-11]. The Danish National Birth Cohort found that excess weight gain in pregnancy increased risks of cesarean delivery and large for gestational age infant [8]. Another US longitudinal cohort study of children 2 to 12 years of age (the National Longitudinal Survey of Youth 1979, NLSY79) found that maternal weight gain during pregnancy ≥20.43 kg were associated with an increased risk of early onset overweight but not late onset overweight [12].

In 2009, the IOM published new recommendations for weight gain during pregnancy [13]. This recommendation varying by pre-pregnancy BMI based on the World Health Organization (WHO) categories was not only suitable for developed countries, but also for Chinese women [14]. It has been shown that being overweight or obese and having a high weight gain, as well as being underweight and having a low weight gain during pregnancy, were associated with increased risks for adverse pregnancy outcomes in women from China and other countries as well [15]. Many studies have suggested that children who gain weight fast in infancy predict a later risk for obesity in childhood and adulthood [16,17]. Two recent researches using the Pregnancy, Infection, and Nutrition Study (2001–2005) reported that infants of women with excessive weight gains had higher weight-for-age, length-for-age and weight-for-length Z scores between birth and 3 years, compared with infants of women with adequate weight gain [18,19]. However, very few studies have examined whether maternal weight gain during pregnancy also predicts weight gain of their children in early infancy. Therefore, the aim of the present study was to evaluate the association of maternal gestational weight gain with anthropometry in the offspring from birth to 12 months old in Tianjin, China.

## Methods

### Study Sample

Tianjin is the fourth largest city with over 12.9 million residents in northern China, and 4.3 million residents live in six central urban districts. Tianjin consists of 16 county-level administrative areas, including six central urban districts, one new urban district, and nine counties that govern towns and rural areas. The prenatal care and children health care in six central urban districts are a routine of a three-tier care system consisting of approximately 65 primary hospitals, 6 district-level Women’s and Children’s Health Centers (also including secondary hospitals), and a city-level (Tianjin) Women’s and Children’s Health Center (also including tertiary hospitals). In Tianjin, all pregnant women are registered at the primary hospitals, and in the 32^nd^ gestational week, they are referred to a secondary hospital or a tertiary hospital for management till delivery. All children are given the health examinations in the newborns, postnatal period, infancy, and at preschool. Tianjin Women and Children’s Health Centre is the leader of the 3-tier care system and responsible for organization, co-ordination and implementation of women and child health care, research and promotion projects. 

Health care records for both pregnant women and their children have been collected and available in electronic form since 2009 [20]. Pregnant Women Health Records start within the first 12 weeks of pregnancy, and include general information (age, occupation, education, date of first visit, numbers of pregnancy/infants, last menstrual period, expected delivery date, smoking habits, etc), history of diseases, family history of diseases, clinical measurements (height, weight, blood pressure, gynaecological examinations, ultrasonography, gestational diabetes (GDM) screening test and other lab tests), complications during pregnancy, pregnancy outcomes (delivery modes, labor complications, etc), and postnatal period examinations (<42 days after delivery). Children Health Records include information from newborns (date of birth, sex, gestational week of birth, birth weight, birth recumbent length, Apgar score, etc), postnatal period (<42 days after birth) (names of the child and his/her parents, family history of diseases, feeding modalities, weight, and recumbent length), infancy (health examinations at each three months during the first 12 months and each six months during 1-3 years), and preschool (health examinations at each year during 3-7 years). The information of feeding modalities (exclusive breast feeding, mixed breast and formula feeding, weaned from breast feeding, and exclusive formula feeding) during the first 12 months, and the measurements of recumbent length/height, weight, blood hemoglobin, and blood pressure (from 3 years old) are collected and available in each health examination. We collected 66,285 records of both mothers and their infants who were born between June 2009 and May 2011. The present study included 38,539 mother-child pairs (60.1%) with all information and clinical measurements after excluding mothers missing the first weight measurement within the first 12 weeks of pregnancy (8,701), multiple births (n=1,496), stillbirth (n=143), premature infants (gestational age <37 weeks, n=1,299), and mother-child pairs missing any variables required for this analysis (n=16,107). Compared with children excluded in the present study, the children included in the present analysis had similar age (12.2 vs. 12.2 months old), were less males (51.5% vs. 53.6%), and their mothers were younger (27.7 vs. 27.8 years old). Of 38,539 mother-child pairs, the rates of child health examination at months 3, 6, 9, and 12 were 88.4%, 90.7%, 94.5% and 98.3%, respectively. The study and analysis plan were approved by Tianjin Women’s and Children’s Health Center Institutional Review Board. Tianjin Women’s and Children’s Health Center has agreed to waive the need for written informed consent from all participants involved in our study because we use the electronic dataset from health care records.

### Measurements

Mothers’ anthropometric data were collected during the pregnancy. Weight and height were measured in light clothing and no shoes using a beambalance scale (RGZ-120, Jiangsu Suhong Medical Instruments Co., China). Blood pressure was measured using a standardized mercury sphygmomanometer (XJ11D, Shanghai Medical Instruments Co., China). Children’s weight and length were measured at birth, 3 months (<4 months), 6 months (≥4 and <7 months), 9 months (≥7 and <10 months), and 12 months (≥10 and <13 months). Weight was measured to the nearest 0.01 kg using a digital scale (TCS-60, Tianjin Weighing Apparatus Co., China). Length was measured to the nearest 0.1 cm using a recumbent length stadiometer (YSC-2, Beijing Guowangxingda, China). We have done a validity study to compare the electronic data of measurements of birth weight and hospitals’ measurements of birth weight among 454 children in six major hospitals. The correlation between two measurements is 0.991. We have also done a validity study to compare the electronic data of measurements of height and weight with the same visit’s measurements of height and weight by trained health workers among 200 pregnancy women and 160 children aged ≤2 years in four different local health centers. The correlations between electronic data and measurement data for body weight are 0.998 for pregnancy women and 0.999 for children, and for height/recumbent length are 0.997 for pregnancy women and 0.999 for children.

Body mass index (BMI) was calculated by dividing weight in kilograms by the square of height in meters. Prepregnancy BMI was categorized as underweight (BMI <18.5 kg/m^2^), normal-weight (18.5 kg/m^2^≤ BMI <24 kg/m^2^), overweight (24 kg/m^2^≤ BMI <28 kg/m^2^), or obese (BMI ≥28 kg/m^2^) using the standard of Working Group on Obesity in China [21]. The Chinese BMI classification standard is used due to the best sensitivity and specificity for identifying risk factors including hypertension, type 2 diabetes, and dyslipidemia in the Chinese population [22-24]. The prepregnancy BMI was calculated using the weight and height recorded at the first prenatal visit within the first 12 weeks of pregnancy. The average (range) gestational weeks at the first weight measurement were 10.4 (2.0-12.9) weeks. The previous study reported that there was a high correlation between self-reported prepregnancy weight and weight recorded at the first visit [25]. Weight gain of mothers during pregnancy was calculated as the difference between prepregnancy weight and delivery weight (end of pregnancy weight). Adequacy of GWG was defined according to the Chinese maternal prepregnancy BMI classification standard and the 2009 IOM GWG recommendations (1): 12.5–18 kg (BMI <18.5 kg/m^2^), 11.5–16 kg (BMI 18.5- 23.9 kg/m^2^), 7–11.5 kg (BMI 24.0–27.9 kg/m^2^), and 5–9 kg (BMI >28 kg/m^2^) [13]. Inadequacy of GWG was defined as below adequacy of GWG and excessive of GWG was defined as above adequacy of GWG according to the Chinese pre-pregnancy BMI classification standard and the 2009 IOM GWG recommendations. We use the translation of U.S. IOM GWG recommendations because no official recommendations exist in China.

We calculated Z scores (standard deviation [SD] scores) independent of sex and age – that is, measurement minus population mean/population SD – in each infant for weight, length, and weight for length and BMI at months 3, 6, 9, and 12 based on the standards for the WHO growth reference [26]. Z scores for birth weight for gestational age, birth length for gestational age, birth weight for length for gestational age, and birth BMI for gestational age were calculated using our own study population means and standard deviations. Since the present study samples were the infants aged under 2 years, BMI normative values are not available [27]. We used the WHO weight for length growth reference. Overweight was defined as a weight for length above the 85th percentile (≥1.035 Z score), and obesity was defined as a weight for length above the 95th percentile (≥1.645 Z score) [26] .

### Statistical analyses

The general characteristics of both mothers and children according to different categories of maternal GWG were compared using General Linear Model and chi-square test. General Linear Models were used to compare the differences in: 1) Z scores for birth body weight, birth body length and birth weight for length; 2) Z scores for body weight, body length and weight for length at months 3, 6, 9, and 12; 3) changes in Z scores for body weight for age, body length for age and weight for length from birth to Month 12 and from Month 6 to Months 12; and 4) changes in Z scores for body weight for age, body length for age and weight for length for each three months, according to different categories of maternal GWG. A repeated measures analysis of variance (General Linear Model) and Least Significant Difference (LSD) post hoc test were used to compare the mean values of Z scores among children who were measured weight and length at each three months during the first year of life (n=25,842) according to maternal prepregnancy BMI and GWG categories. We included 2 models in the analyses: model 1 adjusted for maternal age, maternal height, maternal gestational diabetes (GDM), maternal education, smoking, family income (<2000, 2000-3000, >3000 yuan/month) (US$1=six point one yuan), maternal occupation, and mode of infant feeding; model 2 additionally adjusted for birth variables for gestational age Z score. GDM was diagnosed on the basis of a 50-g 1-hour glucose challenge test (GCT) and a 75-g 2-hour oral glucose tolerance test (OGTT) using WHO criteria [28]. Logistic regression was used to assess the single and joint associations of maternal prepregnancy BMI and GWG with the risk of infant overweight or obesity. The significance of the trend over different categories of maternal prepregnancy BMI and GWG categories was tested in the same models by giving an ordinal numeric value for each dummy variable. The criterion for statistical significance was <0.05 (for two-sided tests). All statistical analyses were performed with PASW for Windows, version 20.0 (Statistics 20, SPSS, IBM, USA)

## Results

The general characteristics of both mothers and children according to maternal GWG categories are presented in Table 1. Mothers who were overweight or obese before pregnancy were older, and reported a lower education level and a lower family income level compared with mothers who were normal weight before pregnancy. Compared with mothers with adequate GWG, mothers with excessive GWG were younger, had a higher prepregnancy BMI, and reported a lower education level, and mothers with inadequate GWG reported a lower education level and a lower family income level. 

**Table 1:** Characteristics of study participants among 38 539 mother-infant pairs according to gestational weight gain categories in Tianjin, China.

	Pre-pregnancy BMI (kg/m^2^)				P for trend	IOM categories §			P for trend
	<18.5	18.5-23.99	24.0-27.99	≥28		Inadequate	Adequate	Excessive	
No. of subjects	4 255	24 678	7 186	2 420		3 793	12 645	22 101	
**Maternal characteristics**									
Gestational weight gain, kg	16.5 (5.1)	17.7 (5.5)	18.0 (6.2)	17.2 (6.9)	<0.001	9.4 (1.9)	14.3 (1.8)	20.9 (5.1)	<0.001
Maternal age before pregnancy, y	26.7 (2.9)	27.6 (3.2)	28.1 (3.5)	28.1 (3.6)	<0.001	27.8 (3.7)	27.8 (3.3)	27.5 (3.2)	<0.001
Gestational age at delivery, wk	39.2 (1.1)	39.3 (1.1)	39.2 (1.2)	38.1 (1.2)	<0.001	39.2 (1.1)	39.2 (1.1)	39.3 (1.1)	<0.001
Prepregnancy BMI, kg/m^2^	17.6 (0.8)	21.1 (1.5)	25.6 (1.1)	30.6 (2.4)	<0.001	20.5 (2.4)	20.9 (2.5)	23.2 (3.7)	<0.001
Mother’s education, %					<0.001				<0.001
University and above	44.8	48.1	41.4	29.3		42.8	49.0	43.7	
Junior college	28.5	26.8	27.8	27.2		25.5	26.1	28.1	
High school and under	26.7	25.1	30.8	43.5		31.7	24.9	28.2	
Family income, yuan/month #, %					<0.001				<0.001
≥3000	55.9	58.3	52.3	43.6		51.6	57.5	55.9	
2000-2999	23.7	21.7	25.1	25.3		23.6	22.4	22.9	
<2000	20.4	20.0	22.6	31.1		24.8	20.1	21.2	
Smoking during pregnancy, %	1.2	1.0	1.2	1.9	<0.001	0.9	0.8	1.4	<0.001
Passive smoking, %	48.3	48.1	50.2	54.5	<0.001	46.5	46.4	50.7	<0.001
Occupation of mother, %					<0.001				<0.001
Farmers and workers	15.9	16.7	19.9	25.1		18.2	16.5	18.3	
Office workers	42.5	42.7	38.9	32.2		40.2	42.7	40.8	
Service professional workers	19.1	21.0	19.6	16.3		18.6	21.3	19.9	
Unemployed persons	10.1	8.4	9.5	12.6		10.5	8.4	9.2	
Other	12.4	11.2	12.1	13.8		12.5	11.1	11.8	
**Child characteristics**					0.159				0.106
Boy, %	50.0	51.6	51.8	52.4		52.2	50.8	51.8	
Mode of infant feeding, %					<0.001				<0.001
Exclusive breast-feeding	15.1	15.3	14.0	11.3		14.9	16.0	14.1	
Mixed breast and formula	68.8	70.8	70.8	68.5		70.5	70.2	70.5	
Weaned from breast-feeding	14.1	12.1	12.6	17.3		12.7	12.0	13.2	
Exclusive formula feeding	2.0	1.8	2.6	2.9		1.9	1.8	2.2	
Weight, kg									
Birth, gram	3265 (388)	3411 (413)	3515 (453)	3594 (489)	<0.001	3275 (399)	3343 (398)	3499 (438)	<0.001
3 month	6.74 (0.77)	6.96 (0.80)	7.04 (0.81)	7.07 (0.84)	<0.001	6.82 (0.79)	6.88 (0.79)	7.02 (0.81)	<0.001
6 month	8.42 (0.96)	8.69 (1.00)	8.82 (1.04)	8.90 (1.07)	<0.001	8.54 (0.99)	8.60 (0.99)	8.77 (1.03)	<0.001
9 month	9.39 (1.03)	9.70 (1.10)	9.87 (1.15)	9.94 (1.18)	<0.001	9.52 (1.09)	9.60 (1.07)	9.81 (1.13)	<0.001
12 month	10.2 (1.1)	10.5 (1.1)	10.7 (1.2)	10.8 (1.3)	<0.001	10.3 (1.1)	10.4 (1.1)	10.6 (1.2)	<0.001
Length, cm									
Birth	50.0 (1.4)	50.3 (1.5)	50.5 (1.5)	50.6 (1.6)	<0.001	50.0 (1.4)	50.1 (1.4)	50.5 (1.5)	<0.001
3 month	62.6 (2.2)	63.0 (2.2)	63.0 (2.3)	63.0 (2.3)	<0.001	62.6 (2.2)	62.8 (2.2)	63.1 (2.2)	<0.001
6 month	68.8 (2.4)	69.2 (2.4)	69.3 (2.5)	69.2 (2.5)	<0.001	68.8 (2.5)	69.0 (2.4)	69.3 (2.4)	<0.001
9 month	73.0 (2.5)	73.4 (2.5)	73.6 (2.6)	73.5 (2.6)	<0.001	73.1 (2.5)	73.3 (2.5)	73.6 (2.6)	<0.001
12 month	76.6 (2.6)	77.0 (2.7)	77.2 (2.7)	77.2 (2.7)	<0.001	76.7 (2.6)	76.8 (2.6)	77.2 (2.7)	<0.001
Overweight at age 12 months*, %	29.2	38.2	44.5	48.4	<0.001	33.1	35.7	41.9	<0.001
Obesity at age 12 months *, %	12.3	18.6	23.5	27.1	<0.001	15.2	17.0	21.4	<0.001

BMI, body mass index.

# US$1=six point one yuan

* Overweight was defined as weight-for-length ≥85th percentile (≥1.035 Z score); Obesity was defined as weight-for-length ≥95th percentile (≥1.645 Z score); Sex-specific weight-for-length percentiles were based on WHO growth reference (World Health Organization, 2006).

§ IOM categories: Inadequate (1): <12.5 kg (pre-pregnancy BMI <18.5 kg/m^2^), <11.5 kg (BMI 18.5- 23.9 kg/m^2^), <7 kg (BMI 24.0–27.9 kg/m^2^), and <5 kg (BMI >28 kg/m^2^); Adequate (1): 12.5–18 kg (BMI <18.5 kg/m^2^), 11.5–16 kg (BMI 18.5- 23.9 kg/m^2^), 7–11.5 kg (BMI 24.0–27.9 kg/m^2^), and 5–9 kg (BMI >28 kg/m^2^); Excessive (1): >18 kg (BMI <18.5 kg/m^2^), >16 kg (BMI 18.5- 23.9 kg/m^2^), >11.5 kg (BMI 24.0–27.9 kg/m^2^), and >9 kg (BMI >28 kg/m^2^), according to the Chinese maternal pre-pregnancy BMI classification standard and the 2009 IOM GWG recommendations.

We compared the mean values of Z scores for body weight, body length and weight for length among children from birth to months 3, 6, 9, and 12 according to maternal prepregnancy BMI and GWG categories (Table S1). Maternal prepregnancy BMI and GWG were positively associated with Z scores for birth weight for gestational age, birth length for gestational age, and birth weight for length. Infants born to mothers with prepregnancy obesity or excessive GWG had greater mean values of Z scores for birth weight for gestational age, birth length for gestational age and birth weight for length, and greater mean values of Z scores for weight for age and length for age at month 3, 6, 9 and 12 compared with other infants born to mothers with prepregnancy normal weight or adequate GWG. The mean values of Z scores for weight for age, length for age, and weight for length in infants born to mothers with prepregnancy overweight or obesity or excessive GWG were significantly higher than those in infants born to mothers with prepregnancy normal weight or adequate GWG, as analyzed by the repeated measures General Linear Model and LSD post hoc test (Table 2 and Table S2). 

**Table 2:** Comparison of Z scores for body weight, body length, and weight for length during the first year of life by the repeated measures.

	Pre-pregnancy BMI (kg/m^2^)				P for trend	IOM categories*#			P for trend
	<18.5	18.5-23.99	24.0-27.99	≥28.0		Inadequate	Adequate	Excessive	
No. of subjects	2 510	14 367	4 070	1 288		2 050	7 268	12 917	
Weight-for-age z-score	0.45 (0.02)	0.74 (0.01)	0.88 (0.01)	0.99 (0.02)	<0.001	0.61 (0.02)	0.69 (0.01)	0.80 (0.01)	<0.001
Length-for-age z-score	0.52 (0.02)	0.69 (0.01)	0.76 (0.01)	0.80 (0.02)	<0.001	0.60 (0.02)	0.65 (0.01)	0.73 (0.01)	<0.001
Weight-for-length z-score	0.25 (0.02)	0.53 (0.01)	0.67 (0.01)	0.79 (0.02)	<0.001	0.43 (0.02)	0.49 (0.01)	0.59 (0.01)	<0.001

Data are means (SE).

Adjusted for maternal age, maternal height, maternal gestational diabetes, maternal education, smoking, family income, maternal occupation, and mode of infant feeding.

* Adjusted also for prepregnancy BMI.

# IOM categories: Inadequate (1): <12.5 kg (pre-pregnancy BMI <18.5 kg/m^2^), <11.5 kg (BMI 18.5- 23.9 kg/m^2^), <7 kg (BMI 24.0–27.9 kg/m^2^), and <5 kg (BMI >28 kg/m^2^); Adequate (1): 12.5–18 kg (BMI <18.5 kg/m^2^), 11.5–16 kg (BMI 18.5- 23.9 kg/m^2^), 7–11.5 kg (BMI 24.0–27.9 kg/m^2^), and 5–9 kg (BMI >28 kg/m^2^); Excessive (1): >18 kg (BMI <18.5 kg/m^2^), >16 kg (BMI 18.5- 23.9 kg/m^2^), >11.5 kg (BMI 24.0–27.9 kg/m^2^), and >9 kg (BMI >28 kg/m^2^), according to the Chinese maternal pre-pregnancy BMI classification standard and the 2009 IOM GWG recommendations.

Table 3 present the changes in Z scores for body weight for age, body length for age, and weight for length among children for each three months from birth to months 12 according to maternal prepregnancy BMI and GWG. After adjustment for maternal age, maternal height, maternal gestational diabetes, maternal education, smoking, family income, maternal occupation, and mode of infant feeding (multivariable model 1), infants born to mothers with prepregnancy overweight and obesity or excessive GWG had the smallest changes in Z scores for weight for age, and length for age from birth to months 3, compared with other infants born to mothers with prepregnancy normal weight or adequate GWG. However, after additional adjustment for Z scores for birth weight for gestational age, birth length for gestational age, birth weight for length, and birth BMI for gestational age in the corresponding analyses (multivariable model 2), infants born to mothers with prepregnancy overweight and obesity or excessive GWG had the greatest changes in Z scores for weight for age and length for age from birth to months 3 compared with other infants born to mothers with prepregnancy normal weight or adequate GWG. From months 3 to 6 and from months 6 to 12, infants born to mothers with prepregnancy overweight and obesity had the greatest changes in Z scores for weight for age and length for age compared with other infants born to mothers with normal weight. From months 6 to 12, infants born to mothers with excessive GWG had the greatest changes in Z scores for weight for age, and weight for length compared with adequate GWG.

**Table 3:** Changes in Z scores for body weight, body length and weight for length from birth to months 3, 6, 9, and 12 according to pre-pregnancy BMI and gestational weight gain categories.

	Pre-pregnancy BMI (kg/m^2^)				P for trend	IOM categories#§			P for trend
	<18.5	18.5-23.99	24.0-27.99	≥28.0		Inadequate	Adequate	Excessive	
**From months 0 to 3**									
No. of subjects	3 331	19 161	5 371	1 696		2 808	9 714	17 037	
Weight-for-age									
Model 1*	0.93 (0.02)	0.88 (0.01)	0.76 (0.01)	0.57 (0.02)	<0.001	0.99 (0.02)	0.92 (0.01)	0.78 (0.01)	<0.001
Model 2†	0.72 (0.01)	0.86 (0.01)	0.87 (0.01)	0.82 (0.02)	<0.001	0.84 (0.02)	0.85 (0.01)	0.84 (0.01)	0.890
Length-for-age									
Model 1*	0.97 (0.02)	0.93 (0.01)	0.85 (0.02)	0.72 (0.03)	<0.001	0.90 (0.02)	0.92 (0.01)	0.90 (0.01)	0.188
Model 2†	0.84 (0.02)	0.92 (0.01)	0.91 (0.01)	0.87 (0.02)	<0.001	0.82 (0.02)	0.88 (0.01)	0.93 (0.01)	<0.001
Weight-for-length									
Model 1*	0.50 (0.02)	0.41 (0.01)	0.32 (0.02)	0.14 (0.03)	<0.001	0.61 (0.02)	0.49 (0.01)	0.30 (0.01)	<0.001
Model 2†	0.21 (0.02)	0.39 (0.01)	0.47 (0.01)	0.48 (0.03)	<0.001	0.41 (0.02)	0.39 (0.01)	0.39 (0.01)	0.730
**From months 3 to 6**									
No. of subjects	3 177	18 214	5 090	1 625		2 654	9 218	16 234	
Weight-for-age									
Model 1*	0.13 (0.01)	0.15 (0.004)	0.18 (0.01)	0.23 (0.01)	<0.001	0.19 (0.01)	0.17 (0.01)	0.14 (0.01)	<0.001
Model 2†	0.11 (0.01)	0.15 (0.004)	0.19 (0.01)	0.25 (0.01)	<0.001	0.17 (0.01)	0.17 (0.01)	0.15 (0.01)	0.037
Length-for-age									
Model 1*	0.06 (0.02)	0.09 (0.01)	0.12 (0.01)	0.11 (0.02)	0.017	0.11 (0.02)	0.10 (0.01)	0.08 (0.01)	0.084
Model 2†	0.05 (0.02)	0.09 (0.01)	0.12 (0.01)	0.12 (0.02)	0.002	0.11 (0.02)	0.10 (0.01)	0.08 (0.01)	0.210
Weight-for-length									
Model 1*	0.29 (0.02)	0.32 (0.01)	0.34 (0.01)	0.41 (0.02)	<0.001	0.33 (0.02)	0.33 (0.01)	0.32 (0.01)	0.381
Model 2†	0.30 (0.02)	0.32 (0.01)	0.33 (0.01)	0.40 (0.02)	0.003	0.33 (0.02)	0.34 (0.01)	0.31 (0.01)	0.189
**From months 6 to 12**									
No. of subjects	3 046	17 354	4 873	1 557		2 559	8 755	15 516	
Weight-for-age									
Model 1*	-0.06 (0.01)	-0.09 (0.01)	-0.07 (0.01)	-0.06 (0.02)	0.027	-0.10 (0.01)	-0.08 (0.01)	-0.08 (0.01)	0.162
Model 2†	-0.08 (0.01)	-0.09 (0.01)	-0.06 (0.01)	-0.03 (0.02)	0.001	-0.12 (0.01)	-0.08 (0.01)	-0.07 (0.01)	0.002
Length-for-age									
Model 1*	-0.24 (0.02)	-0.23 (0.01)	-0.20 (0.01)	-0.16 (0.02)	0.002	-0.19 (0.02)	-0.20 (0.01)	-0.23 (0.01)	0.008
Model 2†	-0.25 (0.02)	-0.23 (0.01)	-0.19 (0.01)	-0.14 (0.02)	<0.001	-0.19 (0.02)	-0.21 (0.01)	-0.23 (0.01)	0.050
Weight-for-length									
Model 1*	0.09 (0.02)	0.07 (0.01)	0.08 (0.01)	0.08 (0.02)	0.418	0.01 (0.02)	0.06 (0.01)	0.09 (0.01)	<0.001
Model 2†	0.10 (0.02)	0.07 (0.01)	0.08 (0.01)	0.08 (0.02)	0.366	0.01 (0.02)	0.07 (0.01)	0.09 (0.01)	<0.001
**From months 0 to 12**									
No. of subjects	3 181	18 206	5 124	1 627		2 686	9 178	16 274	
Weight-for-age									
Model 1*	1.00 (0.02)	0.93 (0.01)	0.88 (0.02)	0.73 (0.03)	<0.001	1.09 (0.02)	1.01 (0.01)	0.84 (0.01)	<0.001
Model 2†	0.75 (0.02)	0.91 (0.01)	1.01 (0.01)	1.03 (0.02)	<0.001	0.90 (0.02)	0.92 (0.01)	0.92 (0.01)	0.395
Length-for-age									
Model 1*	0.80 (0.02)	0.79 (0.01)	0.78 (0.02)	0.66 (0.03)	<0.001	0.83 (0.02)	0.83 (0.01)	0.75 (0.01)	<0.001
Model 2†	0.66 (0.02)	0.78 (0.01)	0.85 (0.01)	0.84 (0.02)	<0.001	0.74 (0.02)	0.78 (0.01)	0.80 (0.01)	0.030
Weight-for-length									
Model 1*	0.88 (0.02)	0.79 (0.01)	0.74 (0.02)	0.61 (0.03)	<0.001	0.96 (0.02)	0.88 (0.01)	0.70 (0.01)	<0.001
Model 2†	0.60 (0.02)	0.77 (0.01)	0.88 (0.01)	0.93 (0.02)	<0.001	0.75 (0.02)	0.78 (0.01)	0.80 (0.01)	0.216

Data are means (SE).

* Model 1 was adjusted for maternal age, maternal height, maternal gestational diabetes, maternal education, smoking, family income, maternal occupation, and mode of infant feeding.

† Model 2 adjusted for above variables and also birth weight for gestational age Z-score in change weight-for-age Z-score, birth length for gestational age Z-score in change length-for-age Z-score, and birth weight for birth length Z-score in change weight-for-length Z-score.

# Adjusted also for prepregnancy BMI.

§ IOM categories: Inadequate (1): <12.5 kg (pre-pregnancy BMI <18.5 kg/m^2^), <11.5 kg (BMI 18.5- 23.9 kg/m^2^), <7 kg (BMI 24.0–27.9 kg/m^2^), and <5 kg (BMI >28 kg/m^2^); Adequate (1): 12.5–18 kg (BMI <18.5 kg/m^2^), 11.5–16 kg (BMI 18.5- 23.9 kg/m^2^), 7–11.5 kg (BMI 24.0–27.9 kg/m^2^), and 5–9 kg (BMI >28 kg/m^2^); Excessive (1): >18 kg (BMI <18.5 kg/m^2^), >16 kg (BMI 18.5- 23.9 kg/m^2^), >11.5 kg (BMI 24.0–27.9 kg/m^2^), and >9 kg (BMI >28 kg/m^2^), according to the Chinese maternal pre-pregnancy BMI classification standard and the 2009 IOM GWG recommendations.

Table 4 showed the relative risks of childhood overweight and obesity at age 12 months stratified by maternal prepregnancy BMI and GWG. After adjustment for all confounding factors, the odd ratio (OR) of childhood overweight or obesity at age 12 months was significantly higher in infants born to mothers with prepregnancy overweight (childhood overweight, OR: 1.30 [95% CI: 1.22-1.38]; childhood obesity, OR: 1.35 [95% CI: 1.25-1.46] ) and obesity (childhood overweight, OR: 1.49 [95% CI: 1.35-1.65]; childhood obesity, OR: 1.63 [95% CI: 1.45-1.84] ) compared with infants born to mothers with normal weight. The higher risk of overweight or obesity at age 12 months was also found among the infants born to mothers with excessive GWG (childhood overweight, OR: 1.29 [95% CI: 1.23-1.36]; childhood obesity, OR: 1.31 [95% CI: 1.23-1.40]) compared with infants born to mothers with adequate GWG. We also analyzed the risk of childhood overweight or obesity according to joint 12 groups of maternal prepregnancy BMI and GWG. When we used infants born to mothers with both prepregnancy normal weight and adequate GWG as the reference, we found that excessive GWG was associated with an increased risk of offspring overweight or obesity at 12 months old in all BMI categories exception for underweight group. 

**Table 4:** Relative risk of childhood overweight and obesity at age 12 months stratified by maternal gestational weight gain and pre-pregnancy body mass index.

	IOM categories#			P for trend	Total
	Inadequate	Adequate	Excessive		
weight-for-length					
Overweight*					
BMI<18.5	0.54 (0.43-0.68)	0.64 (0.55-0.74)	0.75 (0.64-0.89)	0.014	0.63 (0.58-0.68)
18.5≤BMI<24.0	0.91 (0.82-1.02)	1.00	1.12 (1.05-1.20)	<0.001	1.00
24.0≤BMI<28.0	1.08 (0.66-1.79)	1.00 (0.84-1.19)	1.42 (1.31-1.53)	<0.001	1.30 (1.22-1.38)
BMI≥28.0	0.50 (0.14-1.78)	1.11 (0.70-1.77)	1.60 (1.43-1.79)	0.054	1.49 (1.35-1.65)
P for trend	<0.001	<0.001	<0.001		<0.001
Total	0.87 (0.80-0.96)	1.00	1.29 (1.23-1.36)	<0.001	
Obesity*					
BMI<18.5	0.50 (0.38-0.66)	0.54 (0.46-0.65)	0.75 (0.62-0.90)	0.009	0.58 (0.52-0.65)
18.5≤BMI<24.0	0.88 (0.77-1.00)	1.00	1.11 (1.02-1.20)	0.001	1.00
24.0≤BMI<28.0	1.41 (0.79-2.51)	1.19 (0.96-1.47)	1.43 (1.31-1.57)	0.195	1.35 (1.25-1.46)
BMI≥28.0	0.37 (0.05-2.85)	1.31 (0.76-2.26)	1.73 (1.52-1.97)	0.164	1.63 (1.45-1.84)
P for trend	<0.001	<0.001	<0.001		<0.001
Total	0.85 (0.75-0.96)	1.00	1.31 (1.23-1.40)	<0.001	

Data are OR (95% CI).

Adjusted for maternal age, maternal height, maternal gestational diabetes, maternal education, smoking, family income, maternal occupation, and mode of infant feeding.

* Overweight was defined as weight-for-length ≥85th percentile (≥1.035 Z score); Obesity was defined as weight-for-length ≥95th percentile (≥1.645 Z score); Sex-specific weight-for-length percentiles were based on WHO growth reference (World Health Organization, 2006).

# IOM categories: Inadequate (1): <12.5 kg (pre-pregnancy BMI <18.5 kg/m^2^), <11.5 kg (BMI 18.5- 23.9 kg/m^2^), <7 kg (BMI 24.0–27.9 kg/m^2^), and <5 kg (BMI >28 kg/m^2^); Adequate (1): 12.5–18 kg (BMI <18.5 kg/m^2^), 11.5–16 kg (BMI 18.5- 23.9 kg/m^2^), 7–11.5 kg (BMI 24.0–27.9 kg/m^2^), and 5–9 kg (BMI >28 kg/m^2^); Excessive (1): >18 kg (BMI <18.5 kg/m^2^), >16 kg (BMI 18.5- 23.9 kg/m^2^), >11.5 kg (BMI 24.0–27.9 kg/m^2^), and >9 kg (BMI >28 kg/m^2^), according to the Chinese maternal pre-pregnancy BMI classification standard and the 2009 IOM GWG recommendations.

## DISCUSSION

The present study indicated that both maternal prepregnancy overweight/obesity and excessive weight gain during pregnancy were associated with higher birth weight and birth length, and greater weight gain and length gain of their children in the first year of life. Meanwhile, we also found that maternal excessive GWG regardless of prepregnancy BMI was associated with an increased risk of offspring overweight or obesity at 12 months old.

The positive association between maternal excessive GWG and higher birth weight of child was similar to the previous studies [8,15,29]. In addition, we emphatically analyzed the growth speed of infants born to mothers with excessive or inadequate GWG in the first year of life. We firstly explored the potential effects of maternal prepregnancy BMI and GWG on the fatal growth during the first year of life, and found that maternal prepregnancy overweight/obesity and excessive GWG were associated with higher birth weight and birth length, less weight gain and length gain in the first 3 months of life, and more weight gain and greater changes in Z scores for weight for length after 6 months before adjusted for birth variables for gestational age Z score, which was consistent with earlier findings from the PIN 3 Study [18,19]. However, after additional adjustment for birth variables for gestational age Z score, maternal prepregnancy overweight/obesity and excessive GWG were associated with more weight gain and length gain in the first 3 months of life. This suggested that infants born to mothers with prepregnancy overweight/obesity or excessive GWG tended to increase their weight fast from birth. However, it is not clear for the effect of birth size on later growth. Mechanisms that signal and regulate early catch-up growth in the postnatal period may influence the associations between small size at birth and risks for disease in adulthood [30]. Thus, long follow-up of our study will answer this question. 

Our study found that 21.4% of children born to obese mothers were obese (weight-for-length ≥ the 95th sex-specific percentile for age) at 1 year of age, and 17.0% of children born to normal weight mothers were obese. We also found that prevalence of obesity in children born to mothers with excessive GWG was higher than that in children born to mothers with adequate GWG (27.1% vs. 18.6%). These results supported previous studies. A US study reported that 36% of children (3.5 to 4.5 years) of obese mothers were obese, whereas only 7.8% of children of mothers with normal weight were obese [31]. Previous studies have found that offspring born to mothers with excessive GWG or higher prepregnancy BMI were associated with an increased risk of overweight and obesity from childhood to adulthood [32-34]. A cohort study of 10,226 participants from the Collaborative Perinatal Project (1959 -1972) reported a 48% (95% CI: 1.06, 2.06) increased risk of overweight among children at 7 years of age of mothers who gained more than the weight gain recommendations compared with that among the children of mothers who met the weight gain guidelines [33]. Another study found that the risk of childhood (3 to 17 years) overweight for high GWG was 1.16 (95% CI: 1.02, 1.32), whereas for low GWG the risk of childhood overweight was not significant at 1.01 (95% CI: 0.89, 1.15) compared with children of maternal GWG at an average level [34]. We extended this relationship to children at the first year of life. 

We also assessed the joint associations of maternal prepregnancy BMI and GWG with the risk of infant overweight or obesity. The present study found that infants born to mothers with prepregnancy normal weight or overweight/obesity and also with excessive GWG had a higher risk of childhood overweight/obesity at 12 months of age, however, infants born to mothers with prepregnancy overweight/obesity and also with adequate GWG did not have a higher risk of childhood overweight/obesity at 12 months of age, compared with infants born to mothers with prepregnancy normal weight and also with adequate GWG. There are several possible mechanisms responsible for the association between maternal GWG and overweight in the offspring. A “developmental overnutrition hypothesis” could explain this relationship. The hypothesis states that high maternal glucose, free fatty acid, and amino acid concentrations result in permanent changes in appetite control, neuroendocrine functioning and/or energy metabolism in the developing fetus, thus leading to the risk of adiposity in later life[35]. In addition, mothers with excessive GWG may have a high energy diet and low levels of physical activity during their pregnancy and they may pass them on to their offspring. Thus these offspring might be more likely to gain weight in later life. This indicated that maternal excessive GWG might play a more important role in the offspring overweight, and might contribute to the overweight epidemic among infants and children.

There are several strengths in our study, including the use of GWG category instead of net weight gain, and the repeated direct measures of the growth and development of infants at birth and each 3 months until 1 year old. A limitation of our study is that we only followed infant growth to 12 months old. Thus we cannot assess the effect of maternal weight gain during pregnancy on offspring’s growth and development after 12 months old. However, the present study is an ongoing project, and we will get the children’s later growth and development information in the near future. 

In summary, our study indicated that maternal prepregnancy overweight/obesity and excessive weight gain during pregnancy were associated with higher birth weight and birth length, and greater weight gain and length gain in the first year of life. Maternal excessive gestational weight gain regardless of prepregnancy BMI was associated with an increased risk of offspring overweight or obesity at 12 months of age. It is important to pay more attention to maternal influences during pregnancy to prevent the intergenerational cycle of obesity. Strategies to raise public awareness of the risks of maternal adiposity and weight gain during pregnancy on offspring future health are required.

## Supporting Information

Table S1 **Comparison of Z scores for body weight, body length, and weight for length from birth to months 3, 6, 9, and 12 according to pre-pregnancy BMI and gestational weight gain categories.**
(DOC)

Table S2 **Statistical Comparisons of Means and Standard Errors between prepregnancy BMI or IOM categories.**
(DOC)
