# Supplementary material for: Maternal Prepregnancy Body Mass Index and Gestational Weight Gain on Offspring Overweight in Early Infancy
Source: PLoS One. 2013 Oct 11;8(10):e77809. doi: 10.1371/journal.pone.0077809 (PMC3817352; doi:10.1371/journal.pone.0077809)
Supplement: Table S1 — Comparison of Z scores for body weight, body length, and weight for length from birth to months 3, 6, 9, and 12 according to pre-pregnancy BMI and gestational weight gain categories. (DOC) [file pone.0077809.s001.doc]

Table S1. Comparison of Z scores for body weight, body length, and weight for length from birth to months 3, 6, 9, and 12 according to pre-pregnancy BMI and gestational weight gain categories

|  | Pre-pregnancy BMI (kg/m^2^) | | | | P for trend | IOM categories# | | | P for trend |
| --- | --- | --- | --- | --- | --- | --- | --- | --- | --- |
|  | <18.5 | 18.5-23.99 | 24.0-27.99 | ≥28.0 |  | Inadequate | Adequate | Excessive |  |
| Birth for gestational weeks* |  |  |  |  |  |  |  |  |  |
| No. of subjects | 4 255 | 24 678 | 7 186 | 2 420 |  | 3 793 | 12 645 | 22 101 |  |
| Weight-for-gestational age z-score | -0.36 (0.88) | -0.02 (0.94) | 0.25 (1.04) | 0.48 (1.14) | <0.001 | -0.31 (0.91) | -0.17 (0.91) | 0.19 (1.01) | <0.001 |
| Length-for-gestational age z-score | -0.23 (0.90) | -0.02 (0.93) | 0.11 (0.97) | 0.24 (1.05) | <0.001 | -0.16 (0.92) | -0.12 (0.91) | 0.08 (0.97) | <0.001 |
| Weight-for-length z-score | -0.35 (0.89) | -0.01 (0.95) | 0.25 (1.04) | 0.49 (1.13) | <0.001 | -0.31 (0.91) | -0.16 (0.92) | 0.20 (1.01) | <0.001 |
| 3 month |  |  |  |  |  |  |  |  |  |
| No. of subjects | 3 819 | 22 200 | 6 501 | 2 172 |  | 3 360 | 11 361 | 19 971 |  |
| Weight-for-age z-score | 0.60 (0.86) | 0.85 (0.87) | 0.95 (0.89) | 0.98 (0.93) | <0.001 | 0.68 (0.88) | 0.76 (0.87) | 0.93 (0.88) | <0.001 |
| Length-for-age z-score | 0.74 (0.99) | 0.88 (0.99) | 0.90 (1.01) | 0.88 (1.02) | <0.001 | 0.70 (0.97) | 0.79 (0.98) | 0.94 (1.00) | <0.001 |
| Weight-for-length z-score | 0.19 (1.03) | 0.41 (1.04) | 0.52 (1.05) | 0.58 (1.07) | <0.001 | 0.33 (1.04) | 0.36 (1.04) | 0.46 (1.05) | <0.001 |
| 6 month |  |  |  |  |  |  |  |  |  |
| No. of subjects | 3 945 | 22 846 | 6 643 | 2 230 |  | 3 478 | 11 695 | 20 491 |  |
| Weight-for-age z-score | 0.73 (0.91) | 1.00 (0.94) | 1.13 (0.97) | 1.20 (1.00) | <0.001 | 0.85 (0.94) | 0.92 (0.94) | 1.08 (0.96) | <0.001 |
| Length-for-age z-score | 0.81 (1.01) | 0.96 (1.04) | 1.02 (1.05) | 0.99 (1.06) | <0.001 | 0.80 (1.04) | 0.88 (1.03) | 1.02 (1.04) | <0.001 |
| Weight-for-length z-score | 0.48 (1.01) | 0.73 (1.03) | 0.85 (1.05) | 0.97 (1.07) | <0.001 | 0.64 (1.03) | 0.67 (1.03) | 0.79 (1.04) | <0.001 |
| 9 month |  |  |  |  |  |  |  |  |  |
| No. of subjects | 3 560 | 20 447 | 5 989 | 1 999 |  | 3 074 | 10 462 | 18 459 |  |
| Weight-for-age z-score | 0.69 (0.89) | 0.95 (0.93) | 1.10 (0.96) | 1.16 (0.98) | <0.001 | 0.79 (0.94) | 0.92 (0.94) | 1.08 (0.95) | <0.001 |
| Length-for-age z-score | 0.71 (1.00) | 0.87 (1.03) | 0.93 (1.04) | 0.93 (1.05) | <0.001 | 0.73 (1.01) | 0.80 (1.02) | 0.93 (1.04) | <0.001 |
| Weight-for-length z-score | 0.52 (0.97) | 0.77 (1.00) | 0.92 (1.02) | 1.00 (1.03) | <0.001 | 0.64 (1.00) | 0.70 (1.00) | 0.85 (1.01) | <0.001 |
| 12 month |  |  |  |  |  |  |  |  |  |
| No. of subjects | 3 642 | 21 114 | 6 192 | 2 059 |  | 3 208 | 10 738 | 19 061 |  |
| Weight-for-age z-score | 0.68 (0.87) | 0.91 (0.90) | 1.06 (0.94) | 1.16 (0.96) | <0.001 | 0.75 (0.90) | 0.84 (0.90) | 1.01 (0.92) | <0.001 |
| Length-for-age z-score | 0.59 (1.01) | 0.74 (1.04) | 0.82 (1.04) | 0.83 (1.06) | <0.001 | 0.60 (1.02) | 0.68 (1.03) | 0.80 (1.04) | <0.001 |
| Weight-for-length z-score | 0.56 (0.96) | 0.79 (0.98) | 0.95 (1.01) | 1.06 (1.03) | <0.001 | 0.66 (0.99) | 0.73 (0.98) | 0.88 (0.99) | <0.001 |

Data are means (SD).

*Z scores for birth were calculated using our own study population means and standard deviations, and were gestational age and sex specific.

# IOM categories: Inadequate (1): <12.5 kg (pre-pregnancy BMI <18.5 kg/m^2^), <11.5 kg (BMI 18.5– 23.9 kg/m^2^), <7 kg (BMI 24.0–27.9 kg/m^2^), and <5 kg (BMI >28 kg/m^2^); Adequate (1): 12.5–18 kg (BMI <18.5 kg/m^2^), 11.5–16 kg (BMI 18.5– 23.9 kg/m^2^), 7–11.5 kg (BMI 24.0–27.9 kg/m^2^), and 5–9 kg (BMI >28 kg/m^2^); Excessive (1): >18 kg (BMI <18.5 kg/m2), >16 kg (BMI 18.5– 23.9 kg/m2), >11.5 kg (BMI 24.0–27.9 kg/m2), and >9 kg (BMI >28 kg/m2), according to the Chinese maternal pre-pregnancy BMI classification standard and the 2009 IOM GWG recommendations.
