# Supplementary material for: Maternal Prepregnancy Body Mass Index and Gestational Weight Gain on Offspring Overweight in Early Infancy
Source: PLoS One. 2013 Oct 11;8(10):e77809. doi: 10.1371/journal.pone.0077809 (PMC3817352; doi:10.1371/journal.pone.0077809)
Supplement: Table S2 — Statistical Comparisons of Means and Standard Errors between prepregnancy BMI or IOM categories. (DOC) [file pone.0077809.s002.doc]

Table S2. Statistical Comparisons of Means and Standard Errors between prepregnancy BMI or IOM categories#

|  | Mean Difference | Standard Error | P value |
| --- | --- | --- | --- |
| Weight-for-age z-score |  |  |  |
| Pre-pregnancy BMI |  |  |  |
| UW/NW | -0.294 | 0.016 | <0.001 |
| UW/OW | -0.438 | 0.019 | <0.001 |
| UW/OB | -0.541 | 0.026 | <0.001 |
| NW/OW | -0.144 | 0.013 | <0.001 |
| NW/OB | -0.247 | 0.022 | <0.001 |
| OW/OB | -0.103 | 0.024 | <0.001 |
| IOM categories |  |  |  |
| I/A | -0.072 | 0.019 | <0.001 |
| I/E | -0.190 | 0.018 | <0.001 |
| A/E | -0.117 | 0.011 | <0.001 |
| Length-for-age z-score |  |  |  |
| Pre-pregnancy BMI |  |  |  |
| UW/NW | -0.171 | 0.016 | <0.001 |
| UW/OW | -0.245 | 0.019 | <0.001 |
| UW/OB | -0.276 | 0.025 | <0.001 |
| NW/OW | -0.073 | 0.013 | <0.001 |
| NW/OB | -0.105 | 0.022 | <0.001 |
| OW/OB | -0.032 | 0.024 | 0.178 |
| IOM categories |  |  |  |
| I/A | -0.043 | 0.018 | 0.020 |
| I/E | -0.129 | 0.018 | <0.001 |
| A/E | -0.087 | 0.011 | <0.001 |
| Weight-for-length z-score |  |  |  |
| Pre-pregnancy BMI |  |  |  |
| UW/NW | -0.278 | 0.016 | <0.001 |
| UW/OW | -0.419 | 0.019 | <0.001 |
| UW/OB | -0.536 | 0.026 | <0.001 |
| NW/OW | -0.142 | 0.013 | <0.001 |
| NW/OB | -0.259 | 0.022 | <0.001 |
| OW/OB | -0.117 | 0.024 | <0.001 |
| IOM categories |  |  |  |
| I/A | -0.065 | 0.019 | 0.001 |
| I/E | -0.160 | 0.018 | <0.001 |
| A/E | -0.096 | 0.012 | <0.001 |

Pre-pregnancy BMI, UW: underweight; NW: normal weight; OW: overweight; OB: obese.

IOM categories, I: Inadequate; A: Adequate; E: Excessive

# IOM categories: Inadequate (1): <12.5 kg (pre-pregnancy BMI <18.5 kg/m^2^), <11.5 kg (BMI 18.5– 23.9 kg/m^2^), <7 kg (BMI 24.0–27.9 kg/m^2^), and <5 kg (BMI >28 kg/m^2^); Adequate (1): 12.5–18 kg (BMI <18.5 kg/m^2^), 11.5–16 kg (BMI 18.5– 23.9 kg/m^2^), 7–11.5 kg (BMI 24.0–27.9 kg/m^2^), and 5–9 kg (BMI >28 kg/m^2^); Excessive (1): >18 kg (BMI <18.5 kg/m2), >16 kg (BMI 18.5– 23.9 kg/m2), >11.5 kg (BMI 24.0–27.9 kg/m2), and >9 kg (BMI >28 kg/m2), according to the Chinese maternal pre-pregnancy BMI classification standard and the 2009 IOM GWG recommendations.
